# Dissociable Reward and Timing Signals in Human Midbrain and Ventral Striatum

**DOI:** 10.1016/j.neuron.2011.08.024

**Published:** 2011-11-17

**Authors:** Miriam C. Klein-Flügge, Laurence T. Hunt, Dominik R. Bach, Raymond J. Dolan, Timothy E.J. Behrens

**Affiliations:** 1 Sobell Department of Motor Neuroscience and Movement Disorders, Institute of Neurology, UCL, London WC1N3BG, UK; 2 Oxford Centre for Functional MRI of the Brain (FMRIB), Oxford OX3 9DU, UK; 3 Wellcome Trust Centre for Neuroimaging, UCL, London WC1N3BG, UK

## Abstract

Reward prediction error (RPE) signals are central to current models of reward-learning. Temporal difference (TD) learning models posit that these signals should be modulated by predictions, not only of magnitude but also timing of reward. Here we show that BOLD activity in the VTA conforms to such TD predictions: responses to unexpected rewards are modulated by a temporal hazard function and activity between a predictive stimulus and reward is depressed in proportion to predicted reward. By contrast, BOLD activity in ventral striatum (VS) does not reflect a TD RPE, but instead encodes a signal on the variable relevant for behavior, here timing but not magnitude of reward. The results have important implications for dopaminergic models of cortico-striatal learning and suggest a modification of the conventional view that VS BOLD necessarily reflects inputs from dopaminergic VTA neurons signaling an RPE.

## Highlights

► Novel closed-loop (based on neuronal activity) DBS (CL-DBS) compared to standard DBS ► Cortico-pallidal CL-DBS yields greater alleviation of Parkinsonian akinesia ► Cortico-pallidal CL-DBS yields greater reduction of oscillatory neuronal discharge ► Pallido-pallidal CL-DBS leads to dissociation between discharge rate and patterns

## Introduction

Systems-level neuroscience has progressively advanced from descriptive approaches toward those that provide a more mechanistic understanding of the relationship between neural activity and behavior. A paradigmatic example is the characterization of a reward prediction error (RPE) emitted by dopaminergic activity, which provides the strongest link yet between computational explanations of behavior and neural data (Schultz et al., 1997).

RPE theory derives from computational accounts of reinforcement learning that specify how an agent comes to learn the values of different actions and stimuli in a complex environment (Sutton and Barto, 1998). One such account, temporal difference (TD) learning, describes how predictive stimuli are associated with later rewards via the propagation of an error function through successive states, or time steps. This error function, the RPE, reports the difference between observed and predicted rewards at that particular time. Strikingly, recordings from single dopaminergic neurons in the ventral tegmental area (VTA) and substantia nigra pars compacta (SNc) report activity that resembles this precise error function (Schultz et al., 1997; Waelti et al., 2001). Dopamine neurons signal unpredicted rewards but are silent when rewards are fully predicted, instead firing at the occurrence of the earliest predictive stimulus. When an expected reward is omitted, dopamine neurons depress their activity at the precise time that this reward should have occurred. Hence, when stimulus-outcome associations are precise in time, dopaminergic activity, like the TD error function, is precise in time (Hollerman and Schultz, 1998).

By comparison, little is known about dopaminergic activity when the time between predictive event and resulting reward is imprecise. When the occurrence of reward is fully predicted, dopamine neurons show differential firing for equal rewards occurring at different times (Hollerman and Schultz, 1998; Fiorillo et al., 2008). A similar dependence of an RPE on the precise time of reward delivery in the case of unpredicted or partially predicted rewards would have implications for the role of dopamine in learning. More specifically, such a signal would be most relevant in situations where the goal is to learn not only how much, but also precisely when, a reward will ensue.

A temporal dependence for a dopaminergic RPE signal would also have implications for understanding striatal activity as measured by BOLD fMRI, where numerous studies report a correlation between the BOLD signal and RPE in learning studies (O'Doherty et al., 2003; Tobler et al., 2006; Pessiglione et al., 2006; Schönberg et al., 2007; Valentin and O'Doherty, 2009). Although it is possible to detect RPE correlates in the VTA (D'Ardenne et al., 2008), technical limitations imaging this region have meant that it is consistently easier to test for such signals in the striatum. Indeed, a large VTA/SNc projection to the striatum has fostered an implicit assumption that activity here reflects a dopaminergic input (O'Doherty et al., 2004; Campbell-Meiklejohn et al., 2010; and many similar examples).

In fMRI studies, it is often advantageous to introduce significant temporal jitter between events. Whereas some researchers have chosen to eschew this advantage in favor of maintaining temporal precision (Schönberg et al., 2010; O'Doherty et al., 2003; Pessiglione et al., 2006; Gershman et al., 2009; Krugel et al., 2009), others have chosen to maximize BOLD signal sensitivity by introducing significant randomness (up to 10 s) in the interval between conditioned stimulus and outcome (Behrens et al., 2007, 2008; Hare et al., 2008; Cohen et al., 2010; Daniel and Pollmann, 2010). This temporal jitter has in all cases been ignored in the computation of the prediction error, subsequently found to correlate with striatal BOLD signal. Furthermore, even in cases where reward timings are fixed, recent observations suggest that the VS signal codes for a behavioral update as opposed to a value prediction error (Li and Daw, 2011). Consequently, these data raise an intriguing possibility that the striatum encodes a signal that is most relevant to the task at hand, even in situations where this does not correspond to a reward prediction error.

Here, we used BOLD fMRI to test these ideas while human subjects performed a classical conditioning experiment where we introduced two crucial manipulations. First, we compared a situation in which the time-interval between conditioned stimulus (CS) and unconditioned stimulus (US) was fixed, against a situation in which this time-interval was drawn randomly from a learned distribution. Subjects had no influence over the US (reward/no reward) in either type of trial. Second, we included instrumental trials where the subject was asked to guess when the US would be delivered. These were the sole trials where a subject's behavior could influence their eventual payment, but no immediate feedback was given on these trials. Hence, throughout the experiment the relevant variable for optimizing behavior was the timing, and not magnitude of the US. To maximize their accuracy on instrumental trials, subjects had to covertly track US timings during the classical conditioning trials, and compare their internal timing predictions with the experienced US timings. The variable relevant for future behavior was therefore divorced from immediately experienced reward magnitude.

This allowed us to test two independent predictions. We hypothesized that the VTA would code for the time-dependent reward prediction error, as predicted by TD theory. By contrast, because in our task subjects had to learn when, but not how much, reward would occur, we hypothesized that striatal responses would code for timing information, independent of reward, that is informative in subsequent instrumental trials.

## Results

**Figure 1:**
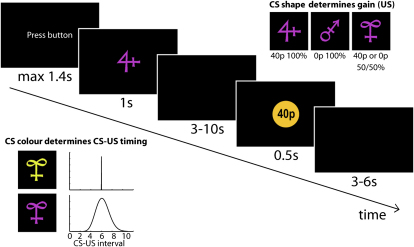
Classical Conditioning Task Dissociates Magnitude and Timing of Reward In each trial, upon the participant's button press, a conditioned stimulus (CS) appeared on the screen and was, after a fixed or variable delay, followed by an outcome of either 40 or 0p (US). In groupU, only 40p outcomes were shown on the screen, 0p outcomes were unsignaled. In groupS, both 40p and 0p outcomes were signaled. The shape of the CS predicted the gain; the color of the CS indicated whether the US would be presented after a fixed delay of six seconds, or a variable delay between 3–10 s. In one out of seven trials (“test trials,” not shown), participants were asked to press a button when they expected the reward to appear. The accuracy of these timing estimates determined the payment and thus, outcome timings but not reward was the variable relevant for future behavior. Behavioral results obtained from test trials are shown in Figure S1.

Thirty subjects (17 females, 20–35 years of age, mean age 26.8 years), of which 28 were included in the analysis (see Experimental Procedures), performed a classical conditioning experiment (Figure 1) while undergoing BOLD fMRI. Subjects were pretrained that three abstract shapes (CS) signaled an outcome (US) of (a), 40p with 100% chance; (b), 0p with 100% chance; or (c), an uncertain outcome of either 40 or 0p with a 50:50 chance. Crucially, the color of the CS indicated whether the US would be delivered after a fixed or variable CS-US interval. Fixed CS-US intervals were always 6 s; variable intervals were drawn from a γ distribution with a mean of 6 s and a standard deviation of 1.5 s (range, 3–10 s). Overall 25% of trials were fixed and 75% of trials were variable. On one trial in seven (randomly interspersed—equally often on fixed and variable timing predicting trials), subjects were asked to press a button at the time they expected the outcome to appear. Subject's accuracy rate at predicting this time (to within 1 s) was multiplied by the free reward they received on all other trials in order to determine their overall final payment. Hence, although positive outcomes were rewarding, it was only through accurately estimating outcome delivery time that subjects could themselves exert a degree of control over their future payment.

### Behavior

Subject's mean time estimate on instrumental test trials was close to the mean CS-US interval of 6 s (6.03 s ± 0.09 grand average over all test trials; 5.85 s ± 0.11 in test trials with variable timing CS; 6.22 s ± 0.09 in test trials with fixed timing CS), showing that participants had acquired an accurate representation of outcome timings and exploited the most rewarding policy. Average timing estimates did not differ significantly from 6 s (p > 0.7 across all test trials).

As expected, in test trials with fixed timing CS, time estimates were less variable than in trials with variable timing CS (Kolmogorov-Smirnov test: p < 0.001, k = 0.23; see Figure S1 available online). Furthermore, time estimates were on average shorter in variable timing compared to fixed timing trials (t_27_ = 5.27, p < 0.001; Table S1).

### VTA Response to Precisely Timed Trials

After careful preprocessing steps to minimize effects of subject motion and physiological artifacts (see Experimental Procedures and Figure S2), we identified a midbrain region in the vicinity of the VTA using a functional contrast. Our aim here was to test whether the VTA BOLD response coded for reward prediction errors in the fixed timing trials, and whether these responses were modulated by outcome time in variable timing trials. Consequently, we chose to identify the VTA using a contrast that was orthogonal to both these effects of interest and in so doing we avoided a potential selection bias. We contrasted unexpected rewards against unexpected zero outcomes in the variable timing trials, averaged across delivery times, in an anatomically restricted region of interest (ROI) around VTA (see Experimental Procedures).

**Figure 2:**
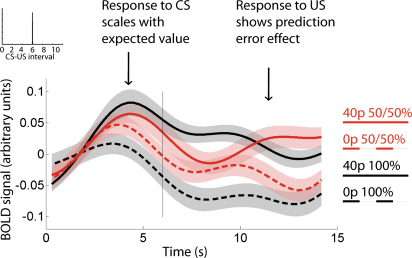
VTA BOLD Response on Fixed Timing Trials Encodes a Standard TD Reward Prediction Error Red lines relate to the same CS condition and differ only at US time. The response to the CS is modulated by expected reward magnitude, the response to the US by the difference between expected and received reward magnitude (prediction error). The US was presented at 6 s (vertical line); shadings indicate SEM. VTA signals were carefully corrected for motion and physiological artifacts (Figure S2).

Using this ROI, we proceeded to test whether the VTA response for fixed trials showed the hallmarks of reward prediction error activity. Consistent with the profile seen in dopaminergic single unit recordings, we found that the BOLD response to the CS increased in proportion to the predicted reward magnitude of the trial (t test on regression slopes: t_27_ = 1.77; p = 0.05; pairwise one-tailed comparisons: 0p versus 0/40p: t_27_ = −2.44, p = 0.01; 0p versus 40p: t_27_ = −4.19, p < 0.001; 0/40p versus 40p: t_27_ = −2.47, p = 0.01), whereas the BOLD response to the US showed a marked increase for unexpected rewards (t_27_ = 4.30, p < 0.001, main effect of 40p US in 50:50 trials), and a difference between unexpected positive and zero outcomes (one-tailed t test: 40p versus 0p US in 50:50 trials: t_27_ = 1.75, p = 0.046; Figure 2).

### VTA Response to Variable CS-US Intervals at US Time

**Figure 3:**
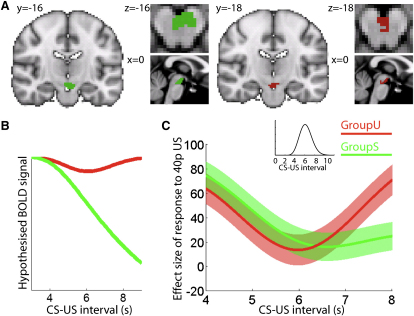
VTA BOLD Response on Variable Timing Trials Encodes a Time-Dependent Reward Prediction Error as Predicted by the Temporal Hazard Function (A) Regions of interest (ROI) in the ventral tegmental area (VTA) for groupS (green) and groupU (red) as defined by a functional contrast between unexpected (50:50) 40 and 0p outcomes in variable timing trials. (B) The predicted BOLD response for variable timing trials was derived from a hazard function, i.e., the probability for a reward to occur at a given time, as predicted by TD theory. Plot shows inverse hazard functions to illustrate the predicted BOLD response to a reward on a 50:50 trial. In groupS all outcomes are signaled; therefore the occurrence of a reward becomes more likely over time, resulting in a predicted decrease of the RPE (shown in green). In contrast, in groupU where zero outcomes are unsignaled, the most likely time for a reward to occur is at 6 s. RPE signals are predicted to be higher when rewards are delivered earlier or later (shown in red). (C) Observed VTA BOLD responses in variable timing trials conformed to TD predictions. Shown are parametric fits of the data (mean ± SEM) at different CS-US intervals. In both groups, an early unexpected reward led to a stronger response than one delivered at the most expected time of 6 s, whereas a late unexpected reward only led to a larger response in groupU. Graphs were obtained by linearly recombining the “constant,” “linear,” and “quadratic” hazard functions (see [B]), with their effect sizes measured from the BOLD data. Raw time courses are shown in Figure S3.

Next, we investigated VTA responses to variable CS-US timings. These should depend on the hazard function, namely, the probability that a reward will occur at a particular time given that it has not already occurred. In order to provide a strong test of this prediction, subjects were divided into two groups. In both groups, 40p outcomes were signaled at the time of delivery. In groupS (signaled group), 0p outcomes were also signaled. Hence, each successive time-step after the CS was more likely to contain an outcome (and thus a reward) as the subject knew that the outcome had not yet been delivered. The hazard function thus increased monotonically through the trial (Figure 3B; inverted function shown in green). In groupU (unsignaled group), 0p outcomes were unsignaled. In this group, the passage of time initially increased the chances of imminent reward (as the peak delivery time approached), and then decreased these chances as it became increasingly likely that the crucial time had passed, resulting in a hazard function that was approximately quadratic and peaking at 6 s (Figure 3B; inverted function shown in red). Because of these group differences in hazard functions, we predicted different BOLD responses to an unexpected reward in the two groups (Figure 3B).

We tested the two hazard functions on the BOLD response to unexpected rewards (for details regarding the general linear model [GLM] see Experimental Procedures). Parameter estimates for both hazard functions were extracted from the VTA ROI. In both groups, VTA data conformed to predictions: the monotonic hazard function predicted data from groupS (t_13_ = 2.60, p = 0.022), and the quadratic hazard function predicted data from groupU (t_13_ = 4.22, p = 0.001), but not vice versa (both p > 0.05; Figure 3C). Furthermore, this difference survived the stringency of a formal between-group comparison (ANOVA group × hazard function, F_1,52_ = 5.18, p = 0.027). Hence, in both groups an unexpected reward delivered early leads to a stronger response than an unexpected reward delivered at an expected time; however, an unexpected late reward only leads to a strong response in groupU, where the temporal hazard function decreases late in the trial. This effect can be seen in the raw BOLD time courses extracted from the VTA, plotted separately for short, middle and long CS-US intervals (Figure S3).

### VTA Response to Variable CS-US Intervals at CS Time

Although we found that the BOLD response to the CS increased in proportion to the expected reward for fixed timing trials, there was no such effect for variable timing trials. There was a general increase in BOLD signal in response to variable cue onset (p < 0.001, t_27_ > 4.0) but this increase did not distinguish between the three reward conditions (p > 0.3; Figure S3). Overall, effects of variable timing cues showed a trend toward being smaller than those of fixed timing cues (t_27_ = 1.99, p = 0.057, comparing responses to any fixed timing CS to those evoked by any variable timing CS), rendering it possible that any effects were too small for such a scaling to be detectable. We note that some TD theories of dopaminergic function make the precise prediction that the cue effect will be diminished under conditions of variable timing (Daw et al., 2006).

### VS Responses

If, in our task, BOLD signals in VS were simply a reflection of VTA output, then this signal should also bear the hallmarks of reward prediction error activity. We therefore first defined a region of interest in VS exactly as we had for VTA, by contrasting unexpected rewards against unexpected zero outcomes in the variable timing trials, averaged across delivery times. Strikingly, in the entire striatum there was not a single voxel that showed a significant increase to an unexpected reward across both groups (Figure S4B). This stood in distinct contrast to the large overlap of significant regions observed in the midbrain (Figure S4A) and was already suggestive of fundamental differences in processing between the two structures, a difference we now examine in detail.

**Figure 4:**
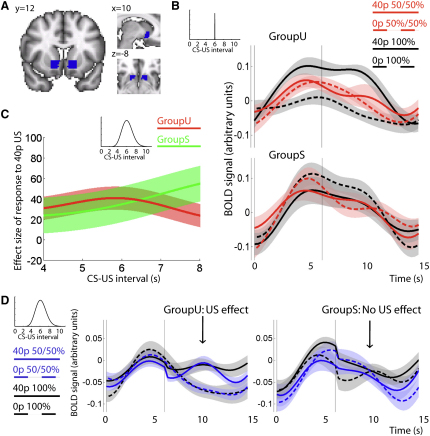
VS BOLD Response Encodes Unexpected Information About Timing, Not Magnitude, of Reward, Inconsistent with a Reward Prediction Error (A) Region of interest (ROI) in ventral striatum (VS) based on the functional response to all fixed timing cues (see Figures S4A–S4D for details on ROI definition). (B) Peri-CS BOLD time courses extracted from the VS ROI for all trials with fixed CS-US interval (top: groupU, bottom: groupS). The response to the CS is modulated by expected reward magnitude in groupU but not groupS. This suggests that VS might in fact code for the expected amount of timing information, the variable relevant for behavior in this task, which stays constant in groupS but not groupU. At US time, responses to unexpected 40p should be significantly larger than those to unexpected 0p for an area encoding an RPE (compare Figure 2 for VTA). This is not the case in VS. Thus, VS responses are not consistent with an RPE at outcome time. (C) In contrast to VTA (Figure 3C), the BOLD response to variable timing trials in VS is not modulated by either hazard function in either group; it remains constant over different CS-US intervals. Raw time courses are shown in Figure S4E. (D) BOLD time courses extracted from VS for all variable timing trials, averaged across all CS-US intervals and aligned to CS (time 0) and US (vertical bar; mean = 6 s). A response to unexpected positive outcomes can be observed in groupU but not in groupS, which is not consistent with TD predictions for an RPE signal. As with the responses observed to the CS (B), this indicates that VS might respond to unexpected information about timing rather than reward. (B), (C), and (D) all denote mean ± SEM.

In defining a VS ROI, we therefore selected voxels that responded significantly to any fixed timing cue, and intersected these voxels with an anatomically-defined VS (Figure 4A, see Experimental Procedures). As was the case in the VTA, this functional contrast was selected to be orthogonal to every test performed in our study, hence eliminating the possibility of selection bias when performing statistical tests within and between regions. Note that the results reported below hold for alternative ROI definitions that are either anatomical or functionally identical across the two structures (see Supplemental Experimental Procedures).

### VS Response to Precisely Timed Trials

Unlike the VTA, our analysis of ventral striatal BOLD signal revealed significant differences between groups in responses to fixed timing trials. In groupU, responses to the CS scaled in proportion to predicted reward magnitude (t test on regression slopes fitted to the responses to a CS predicting 0p, 0/40p, or 40p: t_13_ > 5, p < 0.001), but this was not the case in groupS (p > 0.9). These differences held up when formally comparing the regression slopes between groups within the striatum (two-sample t test: t_13_ = 4.50, p < 0.001), and when comparing between VTA and striatum ROI (ANOVA ROI × group, F_1,52_ = 5.64, p = 0.021). These effects can be seen graphically in Figure 4B. Crucially, in groupU, a 40p reward was the only event that contained information about US timings and consequently a CS that was predictive of the occurrence of reward was also predictive of the occurrence of timing information. By contrast, in groupS, timing information was provided on every trial. Thus, across the two groups, ventral striatal activity was greatest to cues that best predicted information about event timing, whereas VTA activity was greatest to cues that best predicted greater reward.

At the time of the US, there was no evidence for an RPE signal in either group (main effect of 40p US in 50:50 trials: p > 0.7; one-tailed t test 40p versus 0p US in 50:50 trials: p > 0.15; Figure 4B). Formal comparison with the RPE responses observed in VTA in fixed timing trials revealed a 2-way interaction (ROI × 40-versus-0p response: F_1,108_ = 4.58, p = 0.035). The absence of an RPE response was likewise observed in a ROI defined in the dorsal striatum and ventral putamen (see Figures S4F–S4K and Supplemental Experimental Procedures).

### VS Response to Variable Timing Trials

As with the VTA, we next assessed the extent to which ventral striatal BOLD fluctuations to unexpected rewards depended upon a group-specific temporal hazard function. However, in the case of the VS, we could also assess the degree to which unexpected rewards elicited a larger response than unexpected zero outcomes on average across all variable timing trials. We could not perform this analysis for the VTA because this very contrast had been used to define the VTA ROI, and so would be subject to selection bias. In GroupU, where unexpected rewards also carry unexpected timing information, unexpected rewards led to an increase in VS activity (t_27_ = 3.69, p = 0.001, response to 40p versus 0p in 50:50 trials; Figure 4D). By contrast in groupS, where all events carry the same timing information, there was no difference in the average VS responses between rewarded and unrewarded variable timing trials (t_27_ < 1, p > 0.3; Figure 4D). Direct comparison between the effects observed in the two groups showed larger differences between responses to 40p versus 0p in groupU compared to groupS (2-way interaction: group × 40p-versus-0p response: F_1,52_ = 5.18, p = 0.026). Again, whereas the VTA responded to unexpected rewards, the VS responded to unexpected information about event timing.

Furthermore, unlike in VTA, the BOLD signal to unpredictable rewards in variable timing trials did not conform with the group-relevant temporal hazard function (Figure 4C and Figure S4E) (ANOVA group × hazard function, F_1,52_ = 1.68, p = 0.28). Formal comparison with the VTA data revealed a three-way interaction (ROI × group × hazard function, F_1,104_ = 4.72, p = 0.032). The absence of an effect of the temporal hazard function was also true for dorsal striatum and ventral putamen (see Figures S4F–S4K and Supplemental Experimental Procedures). In summary, at US time in variable timing trials, the only event that elicited a significant increase in VS activity was an unexpected reward in groupU—the only event that revealed unexpected timing information.

### Relating VS Responses to Behavior

To examine whether this response to unexpected timing information at US time was related to subject behavior, we performed two further analyses on BOLD responses to unexpected rewards in groupU. We assumed that, in order to perform well on test trials, subjects would covertly time the outcome in each trial. It is therefore conceivable that the VS response to the US in classical conditioning trials might reflect the accuracy of subjects' internal timing estimates and drive behavioral change.

**Figure 5:**
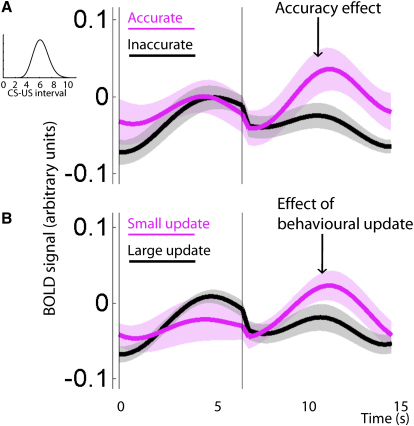
VS BOLD Response Shows Hallmarks of Subject's Covert Timing Behavior (A) The VS response to a US that is unexpectedly close to the subject's average timing prediction from test trials (magenta, accurate) is large compared to that observed to a US that is distant from the subject's average timing prediction (black, inaccurate), analogous to many tasks where unexpectedly successful outcomes lead to large VS responses. Shown is the average BOLD response from variable-timing trials with an unexpected 40p reward in groupU where magenta shows trials in which subject's timing estimates were in the most accurate third, and black any other trials. This effect was not present in VTA, and not present in groupS in either ROI (not shown). (B) In the same trials, VS responses are large, when behavioral updates between two test trials are small, consistent with a behavioral update signal that is informed by the accuracy of subject's covert timing estimates. Similarly, in conventional tasks, subjects are likely to reselect the same option following large VS responses. In (A) and (B), plots show mean ± SEM.

If the VS signal is monitoring task-performance then trials where the subject's prediction is more accurate than expected should elicit a large BOLD response at US time. By analogy, unexpectedly successful outcomes lead to high VS BOLD signal in many tasks. Here, the key measure of success is the subject's accuracy in predicting the US time. To test this hypothesis, we used each subject's mean timing estimate from instrumental test trials as an index of his or her internal prediction of outcome timing. We then examined the classical conditioning trials where the experienced US timing was closest (1/3 trials) to this internal US timing prediction (more accurate trials), and compared VS responses in these trials against those in all other trials (less accurate trials). As predicted, we found larger responses to more accurate trials (t_13_ = 2.76, p = 0.016; Figure 5A). Furthermore, such a signal was not present in the VTA (p = 0.919) and direct comparison between VTA and VS revealed a trend for an interaction (ROI × accuracy: F_1,52_ = 3.57, p = 0.064).

Second, if this VS response is a measure of covert timing performance then, after large VS responses, subjects should not change their timing estimates on subsequent test trials. Again, by analogy to more conventional tasks, high VS BOLD responses are associated with reselecting the same option on the following trial (Li and Daw, 2011). To test this hypothesis, we calculated the change in subjects' timing guesses between one test trial and the next. We then examined VS responses in the classical conditioning trials that occurred between these test trials. Again we examined trials that led to the smallest (1/3 trials) behavioral change (smaller update trials), and compared VS responses in these trials against those in all other trials (larger update trials). As expected, we found larger responses to smaller update trials (t_13_ = 2.20, p = 0.046; Figure 5B). Again, such a signal was not present in the VTA (p = 0.22).

### Ongoing Negative Prediction Errors While Waiting for Reward

Our data show that the BOLD signal from the VTA, but not the VS, is consistent with TD reward prediction errors both to conditioned and unconditioned stimuli. However, in situations with uncertain reward timing, TD theory also predicts that activity in the waiting period between CS and US will be depressed by continual small negative prediction errors, as each successive time bin fails to deliver a reward. This depression should be proportional to the predicted reward level and be more depressed for larger or higher probability predicted rewards.

**Figure 6:**
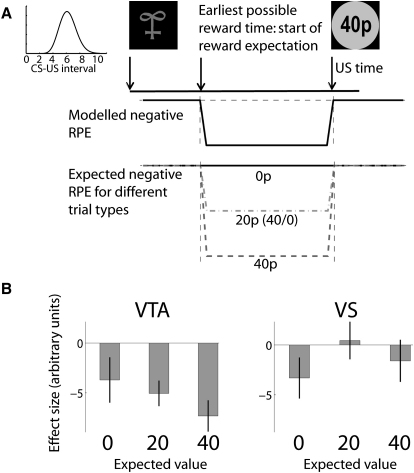
BOLD Response in VTA, but Not VS, Is Depressed in Proportion to Predicted Reward While Awaiting a Reward (A) TD theory predicts continual small negative reward prediction errors (RPE) in all time bins where a reward is expected but fails to occur. This RPE should scale with expected reward. Illustrated are the modeled (middle) and expected (bottom) negative RPEs through the course of a trial (top). (B) Observed effect sizes in VTA conformed to TD predictions and showed larger negative ongoing RPEs when greater reward was expected. No such effect could be observed in VS. Error bars denote SEM.

To examine this hypothesis, we modeled a constant ongoing negative reward prediction error in the time between CS and US in our variable timing trials (Figure 6A). In the VTA, parameter estimates were both negative on average (one sample t test: t_27_ = −4.4, p < 0.001) and exhibited a trend toward being more negative in proportion to the CS reward probability (t_27_ = −1.5, p = 0.08; Figure 6B). Neither of these effects held true in the VS (p = 0.23, 0.75). Formal testing between structures revealed that this ongoing depression of activity was significantly greater in the VTA than the VS (two sample t test: t_27_ = −2.4, p = 0.01), and was modulated by the CS reward probability significantly more strongly in VTA than VS (t_27_ = −2.2, p = 0.02). Hence activity in the VTA alone, but not the VS, conformed with predictions from TD theory at cue time, while waiting for an outcome and at outcome time.

## Discussion

Here, we examined the behavioral and neural effects induced by a task where stimuli were classically conditioned for reward, but where the key variable for behavior was not the receipt of reward but its time of occurrence. We show that activity in the VTA encapsulates RPE predictions derived from TD models. The measured RPE signal in VTA is modulated by the expected reward magnitude but also by the probability of occurrence of a reward at a given time. However, this does not hold true for the VS. VS does not encode a classic TD-RPE; instead, it encodes a task-specific signal reflecting behavioral performance, in the present case, the accuracy of outcome timing predictions. Our findings have important implications for the interpretation of previous studies and for the design of neuroimaging experiments that seek neural correlates of RPEs.

Both single unit (Schultz et al., 1997; Waelti et al., 2001) and fMRI (D'Ardenne et al., 2008) activity report dopaminergic midbrain activity increases to unexpected rewards in a manner consistent with a TD reward prediction error. However, TD theory predicts such activity will be modulated by expectations of when a reward will occur. We formally tested this prediction using BOLD fMRI in conjunction with a conditioning task where the predictability of a CS-US interval was systematically manipulated. When the CS-US interval was fixed and predictable, BOLD activity extracted from a midbrain region corresponding to the anatomical location of the VTA bore all the hallmarks of a reward prediction error signal. When the CS-US interval was varied, BOLD activity was greatest for unpredicted rewards, but this activity was modulated according to a temporal hazard function—the likelihood that a reward would occur at this instance given its prior absence—in agreement with predictions from TD theory (Sutton and Barto, 1998; Daw et al., 2006). Furthermore, as predicted by TD theory (Daw et al., 2006), we show a measurable ongoing decrease in BOLD activity in the same region, when a subject is awaiting the delivery of a reward whose timing is unpredictable.

Crucially, in our study the temporal dependence of BOLD activity cannot be attributed to confounding factors such as waiting costs or temporal discounting of reward. Such arguments might apply to previous studies that have measured the effect of unknown delays on predicted rewards (Roesch et al., 2007; Fiorillo et al., 2008). Here, however, we separated subjects into two groups who encountered identical delays, but different hazard functions. As predicted by Fiorillo et al. (2008), we find it is the temporal hazard function, and not delay costs, that modulate VTA BOLD activity. Notably, BOLD activity in VTA was consistent with a reward prediction error signal, even though the relationship between cues and rewards did not determine behavior.

Insofar as fMRI activity measured in putative VTA reports dopaminergic activity, this finding is of fundamental importance to learning models. Models that consider dopamine as a general teaching signal for cortico-striatal learning (Calabresi et al., 2007; Cohen and Frank, 2009; Reynolds and Wickens, 2002; O'Doherty et al., 2004) should be able to accommodate different responses for rewards that occur at different times, even if the timing information is irrelevant to the learning problem at hand. On initial consideration, the midbrain response we have measured would be most useful for problems where it is important to learn both how much and when reward will ensue.

We report a second set of findings that pertain to the ventral striatal BOLD signal, and its putative relationship with dopamine. The existence of a dense dopaminergic projection to ventral striatum has led to the common assumption that ventral striatal correlates of reward prediction errors simply reflect activity in a dopaminergic input (O'Doherty et al., 2004; Campbell-Meiklejohn et al., 2010, and many similar examples). This view is strengthened by a finding that pharmacological dopamine manipulations have measurable effects on the expression of a ventral striatal reward prediction error (Pessiglione et al., 2006).

Here, however, we describe separable and statistically different patterns of activity between VTA and VS during the course of the same task. This was possible because our task entailed a behavior that was independent of predicted and received reward magnitudes. Subjects were presented with rewards and reward-conditioned stimuli but, unlike in many similar experiments, were not asked to judge how much reward would ensue from each stimulus, or to decide between different stimuli to maximize their reward. Instead, on occasional test trials, they were asked to judge when an outcome would occur. Hence, timing accuracy, not reward, was the variable relevant for behavioral performance. In order to perform well on test trials, subjects had to covertly track outcome timing in normal classical conditioning trials to build an accurate internal timing representation.

At the conditioned cue, BOLD responses in ventral striatum across the two groups reflected not the probability of reward, but rather the probability of timing information being received. At outcome time, activity was largest when new timing information arrived unexpectedly. Furthermore, when such unexpected timing information was received, activity reflected the accuracy of the subject's internal prediction of the event's timing, and the need for behavioral update. Unlike the VTA, in both groups, ventral striatal activity to variably timed outcomes did not reflect the temporal hazard function of reward, and preparatory activity in these trials did not reflect ongoing negative prediction error coding.

Hence, although activity recorded in the putative VTA coded for a reward prediction error even when it did not determine behavior, VS activity at CS and US coded the information about the behaviorally relevant variable—accurate outcome timing predictions.

We note that the findings we present here are not inconsistent with the existence of a VS reward prediction error signal, even a dopaminergic one, in the many situations where subjects' aim is indeed to maximize the occurrence and magnitude of accumulated rewards (Yacubian et al., 2006; Pessiglione et al., 2006; Haruno and Kawato, 2006; Li et al., 2006; Schönberg et al., 2007; Valentin and O'Doherty, 2009). However, our findings can explain why VS reward prediction errors are often not modulated by event-timing, and why they occur in other learning domains. First, when a task requires a subject to accumulate rewards, VS responses to reward do not appear to be modulated by reward delivery time (Gläscher et al., 2010), consistent with the idea that VS encodes signals that are relevant for behavior. Second, again consistent with our data, prediction errors are found to align with the learning dimension of interest in other learning domains. For example, when subjects are asked to learn about reward probability rather than magnitude, ventral striatal activity reflects the occurrence, not the magnitude, of reward (Behrens et al., 2008); this is also true when learning about the probability of aversive events (Seymour et al., 2004; Jensen et al., 2007; Seymour et al., 2007). When subjects learn to predict a sensory event, VS encodes a sensory prediction error (den Ouden et al., 2010), when asked to predict the character or attractiveness of another individual, VS encodes a violation of social expectancies (Klucharev et al., 2009; Harris and Fiske, 2010). It could be argued that this information is transformed into an internal reward (Botvinick et al., 2009), and consistent with that idea, prediction errors can be seen on subject performance (Brovelli et al., 2008; Seger et al., 2010). But even if this interpretation holds in our study, and VS activity is coded in this new “internal reward” frame of reference, it is notable that VTA activity reflects TD prediction errors in the original experimental frame of reference. Thus, a striatal signal that drives behavior coexists simultaneously with a classical reward-based model-free TD signal expressed in the VTA.

## Experimental Procedures

### Participants

Thirty subjects (17 females; 20–35 years of age; mean, 26.8 years) participated in the fMRI experiment and gave informed consent. Subjects were randomly assigned to two groups before the start of the experiment. After exclusion of two subjects (one did not learn the timings crucial for the task as shown in a postscan questionnaire; one was excluded due to excessive head movements: mean estimated displacement >3 cm), both groups included 14 subjects. The study was approved by the local ethics committee.

### Task Design

Three abstract shapes (CS) signaled an outcome (US) of (1), 40p with 100% chance; (2), 0p with 100% chance; or (3), an uncertain outcome of a 50:50 chance of either 40 or 0p. 40p rewards were always signaled by a visual cue. In groupU, 0p outcomes were unsignaled, in groupS, they were signaled by a visual cue. The color of the CS indicated whether the US would appear after a fixed or variable delay. CS-US intervals were 6 s for fixed timing trials. For variable timing trials, we sampled intervals from a gamma distribution with mean μ = 6 s and standard deviation σ = 1.5. Using the equations a = μ^∧^2/σ and b = σ/μ, it follows that a = 24 and b = 0.25. With these parameters, the gamma distribution has values close to zero (<0.01) for x < 3 and x > 10. We restricted our discrete sampling to values in the interval x = [3:10], leading to delays between 3–10 s (Figure 1). Twenty-five percent of trials had fixed timings, 75% of trials had variable timings in order to obtain the same number of fixed, early, middle, and late variable trials.

There were two trial types. Normal classical conditioning trials started with the instruction “Press button” on the screen. Subjects were required to press a button (maximum allowed reaction time: 1400 ms) that brought the CS on the screen (duration: 1050 ms). After the CS-US interval, the CS was, if applicable, followed by a US (duration: 480 ms). The intertrial interval was 3–6 s.

The second trial type, instrumental test trials, looked exactly like normal trials except that the instruction at trial start showed an additional warning “Bucket trial!”. This signaled to subjects that no US would be shown on the screen in this trial, but instead, after CS presentation, subjects would be required to press a second key at the exact time they most expected the reward to occur had this been a normal trial. No feedback was given on these test trials. Subjects were expected to guess the random timing which meant that the optimal strategy was to guess 6 s regardless of condition. Given the distribution of timings, this was the most rewarded policy.

Test trials were randomly interspersed with normal trials but did not occur before the eighth normal trial of each experimental block. On average, there was one test trial for every six normal trials. At the end of each of the four experimental blocks, participants were informed of the number of successful timing predictions in test trials, the total amount of money collected, and the resulting product of the two (corresponding to their payment, see below): “You caught a reward in your bucket in x out of a total of 8 bucket trials. Altogether you collected £y; therefore you won £x/8 ∗ y in this block.”

In total, each subject completed 224 trials, 192 normal trials, and 32 test trials. Normal trials consisted of 144 trials with variable CS-US timing and 48 trials with fixed CS-US timing. This resulted in 36 (12) trials for variable (fixed) timing trials with 100% 40p, 50:50 40p, 100% 0p, and 50:50 0p outcomes, respectively.

### Experimental Procedure

Before entering the scanner, subjects completed a training session consisting of 60 trials. They were instructed to learn how to associate three CS shapes with three possible outcomes (40p, 0p, 40/0p), and two colors with either more or less predictable reward timing. All subjects had learned the associations successfully after the training as shown in a brief questionnaire. However, one subject was excluded because he reported nonexistent changes in color-timing associations after scanning.

The scanning session consisted of four experimental blocks of 48 normal and 8 test trials each. The order of trials was randomized and different in each block.

Subjects were paid according to the number of successful timing estimates given in test trials. More precisely, the sum of all rewards collected during the experiment (amounting to £30 if no trials were missed) was multiplied by the percentage of test trials in which the time they indicated was within 1 s of the true reward time. On average, subjects earned £15 on the task (min £5, max £26), and were paid an extra £10 for their participation.

### Behavioral Analysis

We carried out t tests and Kolmogorov-Smirnov tests on the timing estimates subjects gave in instrumental test trials. Comparisons were done both between and across groups.

### FMRI Data Acquisition

We acquired T2^∗^-weighted EPI images on a 3 T TRIO scanner (Siemens) using a 12-channel head coil. Each of the four blocks consisted of 237 volumes with 43 slices, a 70 ms echo time (TE), resulting in a repetition time (TR) of 3.01 s; the voxel size was 3 × 3 × 3 mm, flip angle −30°. We used a sequence optimized for orbito-frontal and midbrain regions to minimize signal dropout. We also acquired a high resolution structural scan (1 × 1 × 1 mm; 176 partitions, FoV = 256 × 240, TE = 2.48 ms, TR = 7.92 ms, FA = 16°, TI = 910 ms, 50% TI ratio) and a field map (TE1 = 10 ms and TE2 = 12.46 ms, 3 × 3 × 2 mm resolution, 1 mm gap). During scanning peripheral measurements of subject pulse, breathing, and skin conductance responses were made together with scanner slice synchronization pulses.

### FMRI Data Preprocessing

FMRI analysis was implemented using FMRIB Software Library (FSL) (Smith et al., 2004). Data were preprocessed using the default options in FSL: Images were motion corrected (Jenkinson et al., 2002) and unwarped using the acquired field maps. Brain matter was segmented from nonbrain (Smith, 2002) before applying Gaussian spatial smoothing with a 5 mm FWHM kernel. Images were high-pass filtered and registered to the high-resolution structural image (7 degrees of freedom) and then the standard MNI152 template using affine registration (12 degrees of freedom) (Jenkinson and Smith, 2001).

### Maximizing Sensitivity in the Midbrain

Further to using a sequence that minimized signal drop-out in midbrain regions, we performed two steps to increase the sensitivity to BOLD responses in the midbrain. These steps were taken because the anatomical location of the VTA makes BOLD signals in the region exquisitely sensitive to both physiological noise and subject motion. First, we applied conservative independent component analysis (ICA) using MELODIC to identify and remove obvious motion and physiological artifacts (Beckmann and Smith, 2004). Because VTA is especially susceptible to physiological noise, its signal variance was greatly reduced following the removal of noise components (Figure S2A). Second, a physiological noise model was constructed using an in-house developed MATLAB toolbox (Hutton et al., 2011). Models for cardiac and respiratory phase and their aliased harmonics were based on RETROICOR (Glover et al., 2000). The model for changes in respiratory volume was based on (Birn et al., 2006). This resulted in 17 regressors, separate ones for each slice: 10 for cardiac phase, 6 for respiratory phase, and 1 for respiratory volume. We generated these 17 regressors once with respect to every slice (n = 43 slices) to maximize their sensitivity for different slice acquisition times. To match the voxelwise input format required by FSL, each of the 17 regressors was formatted as a four-dimensional volume with identical regressors for voxels within the same slice, but different regressors across voxels of different slices. This resulted in 17 regressors with the following dimensions: 64 (voxels in x) × 64 (voxels in y) × 43 (slices) × 234 (volumes), importantly differing only in the “slice” and “volume” dimensions. Regressors were included in the general linear model (GLM) that led to a further reduction of the signal variance in VTA (Figure S2B).

### fMRI Data Analysis

Temporal difference models predict different patterns of dopaminergic activity in the two groups. For creating the regressors to include in the GLM, we used a hazard function, reflecting the probability that a reward will occur at time *t* given that it has not yet occurred

$$\frac{rP\left(t\right)dt}{\left(1-r\right)+r\left(1-\int_{0}^{t}P\left(t\right)dt\right)},$$

where *P* is a γ distribution with a mean of 6 and a standard deviation of 1.5 from which the CS-US intervals were drawn (Figure 1). We varied the parameter *r* to be *r* = 0.5 to predict the situation when only half of the outcomes were shown (groupU), and *r* = 1 for when all outcomes were shown (groupS). This led to the predictions shown in Figure 3A. In groupU, where the most likely time for a reward delivery is the mean delivery time, the BOLD RPE response is predicted to be large for early and late, but smaller for midtime unexpected rewards. In groupS, it becomes more likely as time passes that each new time bin will contain a reward. The RPE signal is therefore expected to be largest for early, and smallest for late unexpected rewards.

The GLM included 47 regressors in groupS and 39 regressors in groupU. In both groups there were six regressors for CS type lasting for the duration of CS presentation of 1 s [timing (fixed/variable) × expected value (40/20/0p)], eight regressors for positive (40p) outcomes [4 for certainty (100%/50%) × fixed/variable; 4 for certainty (100%/50%) × monotonic/quadratic hazard functions], also modeled with a duration of 1 s, two regressors for the time of CS and response in test trials, again both lasting 1 s, and finally, six regressors produced during realignment for motion correction, and 17 four-dimensional regressors to account for physiological noise. In groupS, the corresponding eight regressors for zero outcomes [4 for certainty (100%/50%) × fixed/variable; 4 for certainty (100%/50%) × monotonic/quadratic hazard functions] were additionally included.

In a second GLM that was otherwise identical, we added six additional regressors modeling a prolonged negative RPE [timing (fixed/variable) × expected value (40/20/0p)], starting 2.5 s after the CS, and ending at the US time or, if no US was presented, 10 s after the CS.

Group analysis was performed using a random effects general linear model (Beckmann et al., 2003; Woolrich et al., 2004).

### ROI Definition and Time Course Extraction

A ROI in the VTA was defined in each group from a contrast comparing variable timing trials with unexpected (50:50) 40 and 0p outcomes, i.e., a contrast with “+1” in the column of the design matrix for unexpected positive and “−1” in the column for unexpected zero outcomes in variable timing trials with uncertain outcome for groupS, and a contrast with just +1 in the column for unexpected positive outcomes in variable timing trials with uncertain outcome for groupU. The ROI included voxels within an anatomically defined region around VTA (coordinates: x: −8 to +6, y: −26 to −14, z: −20 to −12) that reached significance at Z > 2.4 for that contrast in the whole-brain voxelwise analysis.

The VS was defined according to the same procedure but based on the functional response to all CS signaling fixed outcome timing, i.e., a contrast with +1 in the three columns of the design matrix for cues signaling fixed timing (0, unsure, or positive). The VS ROI was restricted to an anatomically defined VS region (coordinates: x: 6 to 18 and −18 to −6, y: 6 to 16, z: −12 to −2). We used the overlap of ROI from both groups in further analyses. All analyses were repeated for two anatomical VS ROIs. A ROI including voxels in the right and left accumbens structures of the Harvard Subcortical Structures Atlas (including probabilities >0.5), and a 5 × 5 × 5 voxel ROI centered at a previously used peak location of (x,y,z) = ±10, 8, −4 (Cools et al., 2002). All statistical tests performed on VS held true for data extracted from these two anatomical ROIs, showing that results did not depend on the exact ROI definition (see Supplemental Experimental Procedures).

BOLD time series for VTA and VS ROI were extracted for each subject by projecting the group ROI back into subject space using the inverse warp field. Time courses were extracted from the preprocessed and ICA-corrected data. The obtained signal was then divided into each trial and resampled with a resolution of 300 ms, with the CS presentation occurring at 0 s. To calculate average BOLD time courses for corresponding trial types, each trial's signal was aligned at the time of CS and US (denoted by vertical lines in the figures), without changing actual timings. When averaging signals separately for trials with short, middle, and long CS-US intervals, the first and last quarter of all time courses were classified as “early” and “late.” This resulted in borders at ∼5 s and ∼7 s for the CS-US interval. For plots of the average BOLD signal, only data points falling in the duration of the mean interval of all averaged time courses were included.

### Statistical Analyses and Figures

We performed t tests and ANOVAs on the parameter estimates obtained from the first-level analyses for the VTA and VS ROI and the effects of interest (e.g., responses to CS and responses to the parametric regressors reflecting the predictions from the hazard functions). To test for an effect of expected reward, slopes were fitted to the estimates of 0p, 20p, and 40p-predicting cues. Similarly in the second GLM, the effect of waiting time was tested by fitting a slope to the estimates from the corresponding 0p, 20p, and 40p regressors. The t tests on slopes were one-tailed as a higher response was expected for higher expected rewards; all other t tests were two-tailed unless indicated otherwise. Where statistical tests involved comparisons against trials in which no event occurred (groupU, no reward trials), group comparisons were performed on the mean time courses as in Behrens et al. (2008).

For the plots comparing predictions from the hazard functions with obtained BOLD responses, parameter estimates of the three resulting contrasts (constant RPE, linear hazard function, quadratic hazard function), multiplied by their parametric modulator, were linearly combined, to obtain the effect size of the RPE across different CS-US intervals (Figure 3C and Figure 4C). Note that these plots do not depict raw BOLD time courses. Peri-CS raw BOLD time courses are depicted elsewhere (Figures S3 and S4E), separately for short, middle, and long CS-US intervals and different CS conditions.

## Supplemental Information

Document S1. Four Figures, One Table, and Supplemental Experimental Procedures
