# Supplementary material for: Dissociable Reward and Timing Signals in Human Midbrain and Ventral Striatum
Source: Neuron. 2011 Nov 17;72(4):654–64. doi: 10.1016/j.neuron.2011.08.024 (PMC3219831; doi:10.1016/j.neuron.2011.08.024)
Supplement: Document S1. Four Figures, One Table, and Supplemental Experimental Procedures [file mmc1.pdf]

Neuron, volume 72

Supplemental Information

Dissociable Reward and Timing Signals in Human Midbrain and Ventral Striatum

Miriam C. Klein-Flügge, Laurence T. Hunt, Dominik R. Bach,

Raymond J. Dolan, and Timothy E.J. Behrens

Inventory of Supplemental Information

Figure 1: Behavioural task; associated with

- **Figure S1**: Distribution of behavioural timing guesses produced on the task
- **Table S1:** Summary of behavioural results produced on the task

Figure 2: VTA BOLD timecourses; associated with

- **Figure S2**: Corrections performed to improve the BOLD signal before extracting timecourses

Figure 3: VTA BOLD response to variable-timing trials; associated with

- **Figure S3**: Underlying raw timecourses from VTA

Figure 4: VS results; associated with

- **Figure S4**: Additional results from VS/striatal ROIs and raw timecourses from VS

Supplemental Figures

Figure S1 shows subject's behaviour on the task depicted in Figure 1

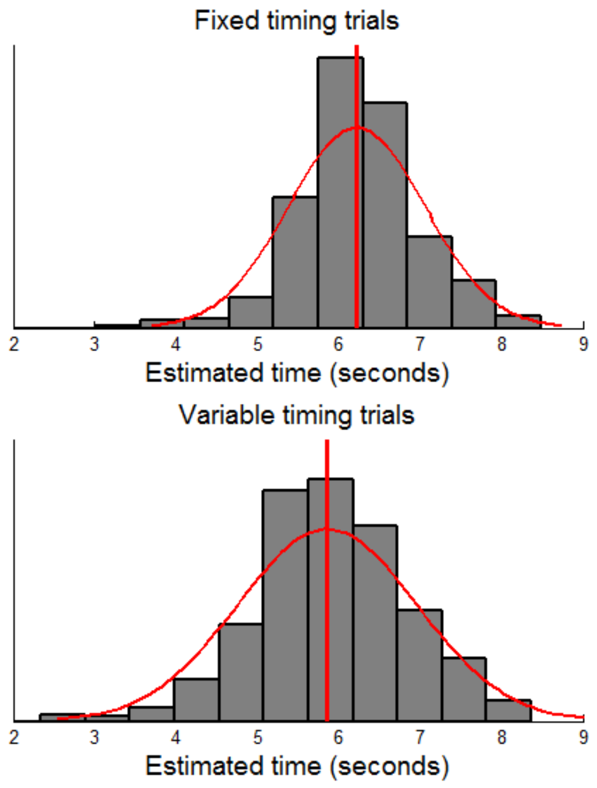

Figure S1

Behavioural results: Shown are the distributions of all subjects' timing estimates on instrumental test trials with fixed timing (top) and variable timing (bottom) predicting CS. In both cases, subjects' estimates are close to the mean CS-US interval of 6 seconds, showing that subjects acquired a good representation of CS-US timings. In variable timing trials, estimates are given earlier and are more variable.

Figure S2 illustrates VTA noise correction methods underlying the data in Figure 2

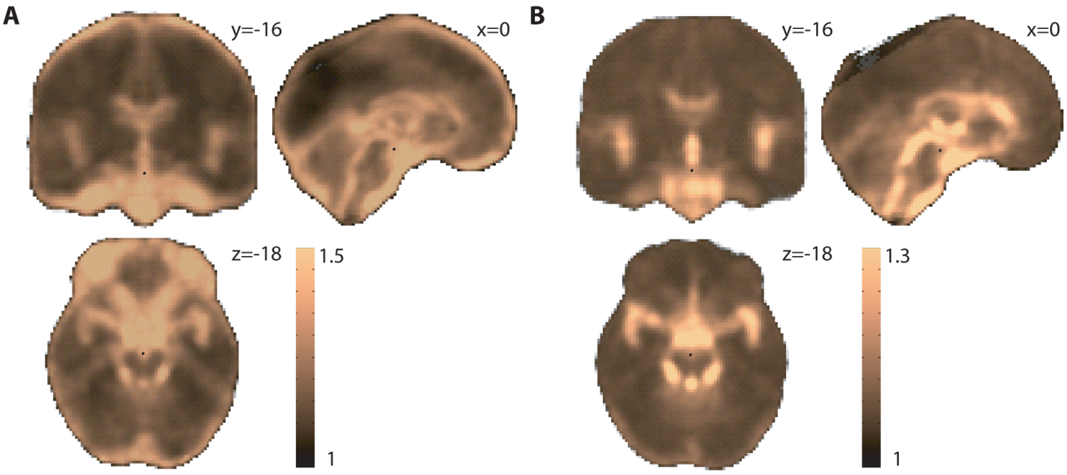

Figure S2

A Independent component analysis (ICA) was used to identify and remove physiological and motion artefacts from the data. This greatly increased the sensitivity to responses from VTA. Illustrated is the ratio of signal variances observed in different regions of the brain before versus after ICA correction (1 (dark): no change, >1 (bright): variance reduced). ICA has removed variance both in areas sensitive to physiological noise, but also near boundaries where subject motion introduces substantial variance. Note how bright the VTA appears in this image in particular. **B** Regressors for breathing and pulse were included in the general linear model which was applied to the ICA-corrected data. This further reduced the variance in mid-brain regions. Colour code as in **A**. VTA coordinates were chosen as in **Figure 3a** and **Figure S3** and are denoted by a black dot.

Figure S3 shows the raw data underlying Figure 3

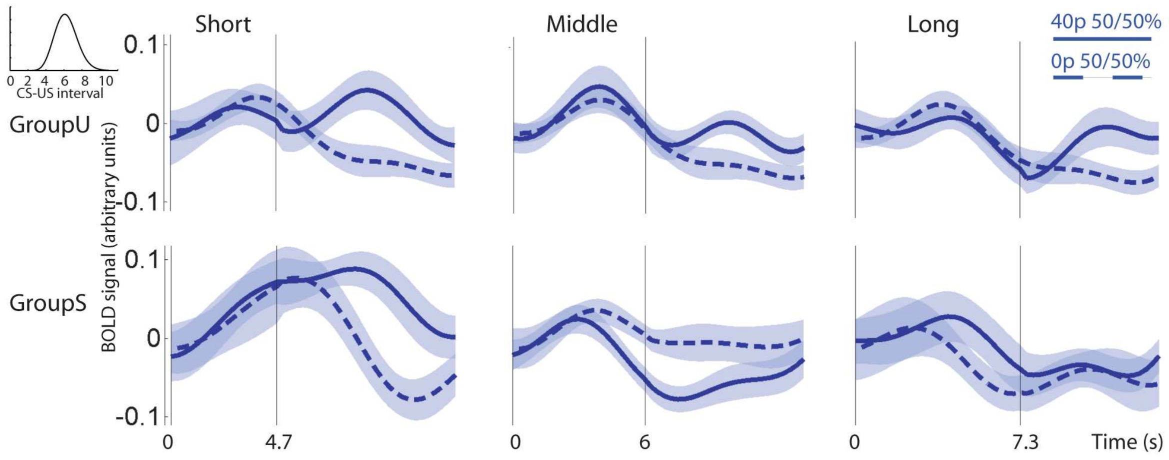

Figure S3

BOLD timecourses extracted from VTA, plotted separately for short, middle and long CS-US intervals for unexpected outcomes (continuous line: rewarded; dotted line: unrewarded; top: groupU; bottom: groupS). The modulations predicted by the hazard function can be observed in the raw data: In both groups, an early unexpected reward leads to a stronger response than one delivered at the most expected time. However, a late unexpected reward leads to a stronger response only in groupU. Shown is the mean ± SEM.

Figure S4 expands on VS responses and dissociations between VTA and VS related to Figure 4

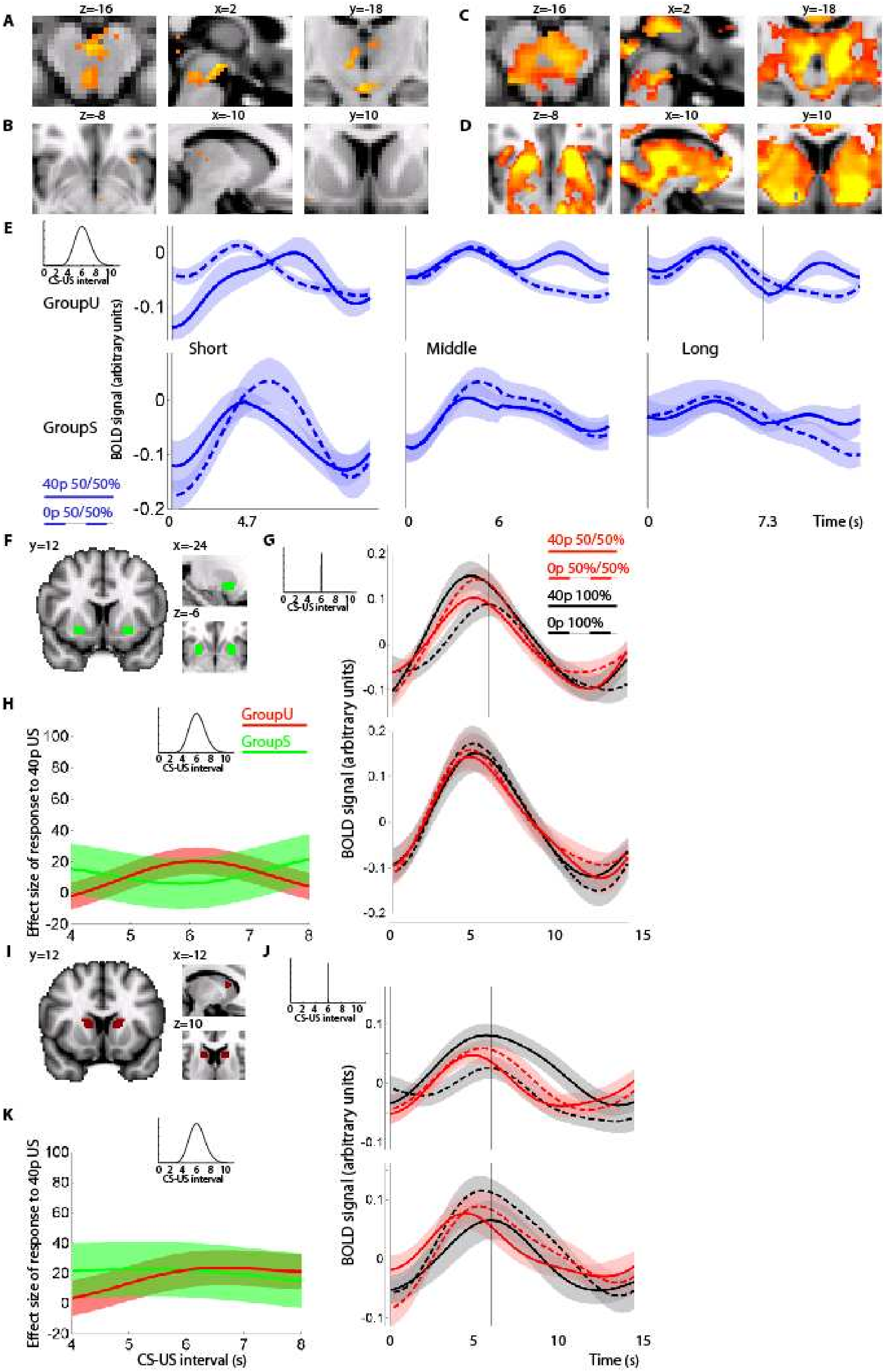

Figure S4

A+B: BOLD response to unexpected rewards in variable timing trials, averaged across delivery times for VTA (**A**) and striatum (**B)**. Not a single voxel shows a significant increase to an unexpected reward across both groups in the striatum, while a large overlap can be observed in the mid-brain. This suggests fundamental differences in processing between the two structures. **C+D**: BOLD response to all CS signalling fixed timing in VTA (**C**) and striatum (**D**); large parts of mid-brain and striatum are active. To maximise selectivity to voxels reflecting dopaminergic processes, the VTA ROI was based on the contrast depicted in **A**. The lack of significant voxels in VS in **B** meant that the VS ROI was based on the contrast shown in **D**. However, all key findings hold true when the VTA ROI is defined based on the contrast depicted in **C**, or when VS is defined anatomically instead of functionally, showing that the results of this study are independent of the method of ROI selection. In **A-D**, the overlap of voxels significant at Z>2.4 in both groups is shown. **E** BOLD timecourses from VS are split by early, middle and late CS-US intervals as in **Figure S3**. The modulation over time observed in VTA is not present in VS. **F-K** BOLD timecourses extracted from a ROI in the ventral putamen (F-H) and dorsal striatum (caudate; I-K) are shown as in **Figure 4**. The ROIs were defined based on previously reported coordinates. As observed in ventral striatum, BOLD responses in ventral putamen and dorsal striatum also do not encode a reward prediction error, and they are not modulated by the temporal hazard function in either group. **E, G, H, J, K** all show mean ± SEM.

Table S1 summarizes behavioural performance on the task in Figure 1

**Timing estimates in test trials (seconds)**	**All test trials**	**Fixed timing CS**	**Variable timings CS**
**GroupS, n=14**	6.05 ± 0.11	6.25 ± 0.10	5.85 ± 0.13
**GroupU, n=14**	6.02 ± 0.16	6.19 ± 0.15	5.85 ± 0.18
**Both groups, n=28**	6.03 ± 0.09	6.22 ± 0.09	5.85 ± 0.11

Supplemental Experimental Procedures

Alternative ROI definitions

Whilst there was no statistical bias introduced by our selection method for neither VTA nor VS ROIs, readers might nevertheless question the choice of different methods of ROI definition in the two different structures. As explained in the main text, and shown in Figures S4, A-D, we could not define the VS using a reward sensitive contrast as it was not reward sensitive in our task. An alternative would be to define the VTA using a cue-sensitive contrast. We decided against this approach because a large region including the VTA and neighbouring structures show cue-sensitivity (**Figure S4C**), whereas a much more focal region in the immediate vicinity of the VTA is reward sensitive (**Figure S4A**). A reward sensitive contrast was much more likely to reflect activity derived from the VTA. However, as shown below, the statistical dissociations that we report are present with either method of VTA definition.

A second alternative would be to define both structures anatomically. We found that all effects reported for VS remain significant if the VS ROI is defined anatomically based on the Harvard Subcortical Structures Atlas, or based on previously reported coordinates (see below). However, for the VTA, an anatomical ROI definition is not straight-forward. There are two reasons for this. First, it is a very small structure even on an anatomical image. Second and most importantly, it is placed in the brain in a region that is one of the least likely to co-localize between functional and structural scans. As its position is displaced by susceptibility induced distortions, we acquired field maps to correct this distortion within the limitations of current methodology. Nevertheless, we acknowledge it is unlikely that an anatomical ROI will be as sensitive as a functional localizer.

Additional VTA ROIs

Here, we re-performed all statistical tests in two different ways, both of which define the VS and VTA based on the same contrast.

An alternative VTA ROI was defined using the response to all fixed timing cues, the contrast used for the VS ROI in the main manuscript. We extracted all voxels at $Z>2.4$ (equivalent to the ROI

definition of VS) and redid all statistical tests on the data extracted from this new ROI. In both groups VTA data still conformed to TD predictions: the monotonic hazard function predicted data from groupS ($p=0.019$; originally $p=0.022$), and the quadratic hazard function predicted data from groupU ($p=0.030$; originally: $p=0.001$), but not vice versa (both $p>0.05$). This difference survived the stringencies of the between-group comparison as a trend (ANOVA group × hazard function $p=0.09$; originally $p=0.027$). The 3-way interaction comparing VTA and VS data reproduced the effect originally reported (ROI × group × hazard function, $p=0.030$; originally 0.032). To further demonstrate the robustness of the reported effects, a second new VTA ROI was extracted from the response to all fixed timing cues, this time including all voxels at $Z>3$. The four effects reported above yielded $p=0.015$ for groupS, $p=0.035$ for groupU, $p=0.079$ for the 2-way interaction, and $p=0.021$ for the 3-way interaction. These tests therefore replicate the original results and show that there is no possibility that they are a function of method of defining the ROI.

Additional VS ROIs

To show that the absence of an RPE response in VS was not caused by the exact way of ROI definition, two alternative VS ROIs were defined anatomically based on the Harvard Subcortical Structures Atlas (VS2), and previously reported coordinates (VS3). Statistical tests that had shown RPE responses in VTA were repeated for these two anatomical VS ROIs. Original VS results are reported for comparison.

VS response to precisely timed trials

In groupU, responses to the CS scaled in proportion to predicted reward magnitude (t-test on slopes fitted to the responses to a CS predicting 0p, 0/40p, or 40p: VS, VS2, VS3 all $p<0.001$), but this was not the case in groupS (VS, VS2, VS3 all $p>0.9$). These differences held up when formally comparing the reward slopes between groups within the striatum (2-sample t-test: VS: $p<0.001$; VS2 and VS3: $p=0.001$), and when comparing between VTA and striatum ROIs (ANOVA ROI × group, VS: $p=0.021$; VS2 and VS3 both $p=0.023$). At the time of the US, there was no evidence for an RPE signal in either group (main effect of 40p US in 50:50 trials: VS, VS2, VS3 all $p>0.7$, one-tailed t-test:

40p vs. 0p US in 50:50 trials: VS, VS2, VS3 all p>0.15, **Figure 4**). Formal comparison with the RPE responses observed in VTA in fixed timing trials revealed a 2-way interaction (ROI × 40-vs-0p response: VS: p=0.035, VS2: p=0.027, VS3: p=0.013).

VS response to variable timing trials

Furthermore, unlike in VTA, the BOLD signal to unpredictable rewards in variable timing trials did not conform with the group-relevant temporal hazard function (**Figure 4C and S4E**; ANOVA group × hazard function: VS: p=0.28; VS2: p=0.31; VS3: p=0.76), with neither hazard function making a significant contribution to the BOLD signal from either group (VS: p>0.2; VS2: p>0.13; VS3: p>0.3). Formal comparison with the VTA data revealed a 3-way interaction (ROI × group × hazard function, p=0.032; VS2: p=0.039; VS3: p=0.098).

Dorsal striatum and ventral putamen

Previous studies report prediction error responses in different parts of the striatum, such as ventral putamen (O'Doherty et al., 2003; O'Doherty, 2004) and dorsal striatum (e.g., Schonberg et al., 2007). To show that the absence of an RPE response was not specific to the part of ventral striatum centering on nucleus accumbens, we defined two additional ROIs in ventral putamen and dorsal striatum using the same procedure as for VS (i.e. including voxels responding to a CS signalling fixed outcome at Z>2.4; **Figure S4, F-K**). ROIs were anatomically constrained based on coordinates previously reported for these structures (O'Doherty et al., 2003; O'Doherty, 2004; Schonberg et al., 2007). For ventral putamen, all significant voxels within the anatomical region [x: ±20 to ±28, y: 2 to 14, z: -10 to -4] were included in the ROI, which included the previously reported coordinates [-27, 3, -9] and [-27, 15, -3] (O'Doherty et al., 2003) and [-26, 8, -4], [-28, 8, -6], [26, 6, -8], and [20, 12, -8] (O'Doherty, 2004). This region did not overlap with the original VS ROI (**Figure S4, F-H**). For the dorsal striatum, we included any voxel in the range [x: ±10 to ±18, y: 10 to 16, z: 6 to 14] (**Figure S4, I-K**), which was based on Schonberg et al.'s coordinates (2007; [18 15 15] and [18 12 15]).

Neither ventral putamen, nor dorsal striatum encoded an RPE. BOLD responses to the CS did not reflect the expected reward magnitude in groupS (VS, putamen and dorsal striatum all $p>0.9$), and responses in groupU reflected the amount of expected timing information to a weaker extent than in VS (VS: $p<0.001$; putamen: $p=0.02$; dorsal striatum: $p=0.002$). Inconsistent with an RPE response, there was no significant difference between responses to unexpected 40p and 0p outcomes, and no main effect of unexpected 40p outcomes (both $p>0.2$ for all ROIs). As for VS, there was also no effect of the temporal hazard function (putamen/dorsal striatum: groupU and groupS for both hazard functions $p>0.2$). Crucially, neither region showed the modulation of activity by accuracy of timing prediction that **was** present in the VS (for groupU: VS: $p=0.016$, putamen: $p=0.48$, dorsal striatum: $p=0.28$).

In summary, RPE responses observed in VTA were absent in dorsal striatum and ventral putamen, and effects reported for VS were weaker or absent in these two structures.

Behaviour in groupS versus groupU

To show that the absence of an RPE effect in VS cannot be due to a lack of learning in one of the groups, we directly compared the performance on test trials across groups. The number of successful timing predictions on test trials did not differ between groupU and groupS ($t_{13}=1.92$, $p=0.14$), and there was no difference in average precision (two-sample t-tests on (a) all timing estimates: $t_{26}=-0.09$. $p=0.93$; (b) fixed timing estimates: $t_{26}=0.29$, $p=0.78$; (c) variable timing estimates: $t_{26}=-0.35$, $p=0.73$). All results described for the combined data of both groups held true when examining each group on its own (Kolmogorov-Smirnov test: $p<0.001$ for both groups; average estimates non-significantly different from 6s: $p>0.5$ in both groups). Thus, results in VS cannot be explained by poor learning of CS-US intervals in either group.
